# Correlation between moral sensitivity and self-esteem in nursing personnel

**Published:** 2017-12-30

**Authors:** Farideh Rahnama, Marjan Mardani-Hamooleh, Marjan Kouhnavard

**Affiliations:** 1 *MSc, Education Development Center, Saveh University of Medical Sciences, Saveh, Iran. *; 2 *Assistant Professor, Department of Psychiatric Nursing, Iran University of Medical Sciences, Tehran, Iran. *; 3 *Children’s Medical Center, Tehran University of Medical Sciences, Tehran, Iran.*

**Keywords:** *Moral sensitivity*, *Nurse*, *Nursing ethics*, *Self-esteem*

## Abstract

Nurses are continuously involved with ethical problems in their area of practice and need to possess a satisfactory level of moral sensitivity in order to be able to offer moral care. Additionally, they act as agents for proper management of ethical dilemmas and are therefore required to have high self-esteem. This study aimed to determine the correlation between moral sensitivity and self-esteem in nurses. In this descriptive-correlational research, sample study included 204 nursing personnel working in hospitals affiliated with Iran University of Medical Sciences. Participants were selected by convenience sampling. The data were collected using a demographic form, Lützén’s Moral Sensitivity Questionnaire, and Rosenberg’s Self-Esteem Questionnaire. Then, the data were analyzed using descriptive and analytical statistics. Written informed consent was obtained from each subject who participated in the research. The mean score for moral sensitivity of the samples was 69.15 ± 5.70, and 20.01 ± 4.76 for their self-esteem. Pearson’s correlation coefficient test indicated a meaningful and positive relationship between the two variables under study (r = 0.472 and *P* = 0.001). There was no correlation between the participants’ demographic data and moral sensitivity (*P *> 0.05), but a significant relationship was found between the participants' level of education and the variable self-esteem (*P* < 0.05). Since there was a positive and significant relationship between moral sensitivity and self-esteem among the nursing staff, nursing managers should focus on improving the quality of patient care by promoting nurses’ moral sensitivity inspired by high self-esteem.

## Introduction

The ethical principles shape the ethical framework emphasizing what counts as true or false according to a set of rules and behaviors considered as right or wrong. Ethics is a spawn of values formed by human beings. Moral sensitivity, on the other hand, is an individual’s skills and ability to interpret the reactions and feelings, take the necessary actions or decisions, examine the effect of possible consequences on the people, and accept responsibility for these decisions, actions and outcomes (1). 

As a matter of fact, nurses are invariably bound up with ethical concepts and problems in their work environment. Among the ethical challenges that nurses face are issues such as working with patients at the end of life, observing patients’ suffering and illness, lack of organizational support, and working stints to meet organizational demands (2). Therefore, it is essential for them to bring the concept of moral sensitivity into focus because their ethical performance in providing patients with nursing care depends on it (3). On the other hand, nurses encounter a broad range of ethical ambiguities as they go about their professional interactions with others. How they behave under these conditions as well as a plethora of professional decision-making shows that they require moral sensitivity in order to properly work these ambiguities out; otherwise, they would act inefficiently in difficult situations when providing care to patients (4). However, the current research results suggest that moral sensitivity in nurses is at a moderate level of 42 - 58% (4 - 6).

The findings of Iranian studies shows that high moral sensitivity among nurses increases nursing leadership and reduces moral distress (7, 8). These findings emphasize the importance of addressing the issue of moral sensitivity in nursing practice and the fact that it ensures a wide range of positive ethical implications in the profession. 

Now the question that comes to mind is: What components could potentially be associated with nurses’ moral sensitivity? One of the variables that seems to have potential in this regard is self-esteem. It is a personality trait that plays an important role in the mental health of individuals. Self-esteem is a measure of approval, acceptance, and a feeling of self-worth toward oneself. There is an undeniable relationship between self-esteem and the ability to establish interpersonal contacts as an integral component of the effective care process (9). Relevant studies conducted in the nursing context show that self-esteem of 46 - 55% of nurses is at an intermediate level (10 - 13). High self-esteem facilitates interpersonal relationships and has a positive effect on the thinking, feeling and practice of nurses in providing care. Nurses with high self-esteem are capable of providing better care services to patients and less likely to suffer burnout (14). Additionally, these nurses consider the changes that creep into their career, such as structural reforms that lead traditional nursing to teamwork nursing, as fruitful challenges (15). On the other hand, the lower nurses’ self-esteem is, the lesser their professional success will be (9). Nurses with low self-esteem consider changes that occur in their career as challenges that impose colossal pressure while wreaking havoc on them (14). Nurses must therefore beware of factors that reduce their self-esteem while providing moral care. Thus, moral sensitivity is considered as a feature that causes nurses to fully recognize the ethical challenges of the clinical environment and have a good understanding of their work situation. Therefore, by controlling the clinical challenges faced by nurses, the pressure that is imposed on them due to low self-esteem will also be reduced. This emphasizes the need to examine the relationship between the two variables under study even more closely.

It should be acknowledged that ethics is an integral part of nurses’ professional life. It lends sense to their working lifestyle. Nurses are in long-term contact with patients and other healthcare staff. Therefore, the shadow of ethics should be constantly extended to all their professional practices. Accordingly, it is important to consider the various ethical dimensions of the nursing profession, as neglecting this part of nurses’ professional life will cause irreparable harm (16). On the other hand, since nursing ethics encompasses a wide area, a comprehensive look at ethical concepts would reveal the possible relationship between these and other concepts, including psychological variables. Utilizing an interdisciplinary approach will help nursing ethics specialists enhance the richness of this area. Therefore, given that moral sensitivity is a cornerstone of professional ethics in nursing (17), and self-esteem as a personality trait affects nurses’ professional performance, the investigation of the relationship between these two variables is essential.

As agents for the correct management of ethical issues, nurses seem to require both moral sensitivity and high self-esteem. Considering the ethical problems encountered by nurses in hospitals and the different attitudes that they adopt, as well as the importance of high self-esteem in the profession, it may be concluded that perhaps there is a correlation between the two variables of moral sensitivity and self-esteem. Studies have already been carried out throughout the world to examine these two variables separately or against other variables, but the researchers could not access any studies that have directly investigated the relationship between them. However, several studies have been conducted in Iran and elsewhere that examined each of these two variables alone or in relation to other variables.

Therefore, considering the importance of the subject and the effect of these variables on the behaviors of nurses as well as their potential impact on the quality of nursing care, the present study aimed to determine the correlation between moral sensitivity and self-esteem in nurses.

## Method

This study is a descriptive-correlation research. In the beginning 3 hospitals affiliated to Iran University of Medical Sciences were chosen by simple random method in 2016. The study population consisted of all the nursing staff employed in these hospitals, which by implication included all the nurses of the selected hospitals with a minimum of one-year work experience. 

It should be noted that due to the nature of the concept of moral sensitivity in nursing, the employees experience it as a result of working in the clinical environment; therefore, the researchers considered at least one-year work experience as the criterion for the participants to enter the study. Other Iranian studies on moral sensitivity in nurses have also considered one year as the minimum work experience for selection of their participants (2, 7).

Participants were selected by convenience sampling. The sample size was estimated at 200 for the confidence interval of 95%, and power to test 80%, assuming the correlation coefficient between moral sensitivity and self-esteem among the nurses was 0.2, so that the relationship between the two variables could be considered statistically significant. Given the possibility of a lack of cooperation on the part of the study samples, 220 nurses were selected for the research. It should be added that 9 subjects opted out of the study for personal reasons, and 7 questionnaires were defaced. As a result, this study was conducted with the participation of 204 nurses.

In this study, ethical approval was first received from Saveh University of Medical Sciences (IR.SAVEHUMS.REC.139506), and the necessary coordination with hospital managements was then achieved through a letter of introduction from the university. Written informed consent was obtained from each subject prior to participation in the research. Furthermore, the participants received the necessary information on the research process, and they were assured that their participation in the research was completely voluntary and they had complete freedom in this regard as well. In addition, the participants were assured that their information would be protected and confidentiality of the data would be maintained. 

The researchers collected the data over the course of 3 months by meeting the participants in their working departments. A demographic information form, a moral sensitivity questionnaire, and a self-esteem questionnaire were used for data collection. The demographic information form examined age, gender, marital status, work experience, education level, shift work and the workplace ward of the participants. The moral sensitivity questionnaire was constructed by Lützén et al. in 1994 and contained 25 questions (18). This questionnaire measures moral sensitivity on a five-level Likert scale: strongly agree, relatively agree, relatively disagree, strongly disagree, and neutral, each receiving a score of 0, 1, 2, 3, and 4 respectively. The total scores range from 0 to 100. Scores of 0 - 50, 50 - 75 and 75 - 100 represent low, moderate and high levels of moral sensitivity respectively. This questionnaire has been used in a variety of researches on nurses and its validity and reliability have been confirmed (4 - 5, 18).

The self-esteem questionnaire was designed by Rosenberg in 1965 (19), and consisted of 10 items on a 4-point Likert scale with the following response options: fully agree, agree, disagree and strongly disagree, scored from 0 to 3. The overall scope of the score was 30; scores higher than 25 indicated high, 15 - 25 moderate, and less than 15 low self-esteem. This questionnaire has also been used in many studies on nurses and its validity and reliability have been confirmed (11, 20, 21).

Iranian studies reported the questionnaire reliability 80- 83% using the Cronbach's Coefficient Alpha (22, 23). Validity and reliability of both questionnaires were confirmed by the researchers using content validity and test-retest methods, respectively. In order to obtain content validity, the viewpoints of 7 professors of the nursing faculty of Iran University of Medical Sciences were used and their corrective comments were applied in the questionnaires. It should be noted that in order to adapt the English version of the tools to their Persian format, in addition to the Persian version previously used by Iranian scholars, the English version of the tools was also available to the above-mentioned professors. The reliability of the questionnaires was confirmed by test re-test in two weeks by participation of 10 nurses*.* The results of the test (2 times) using the Pearson’s correlation coefficient were calculated to be 0.92 for the moral sensitivity questionnaire and 0.88 for the self-esteem questionnaire. 

Statistical data analysis was performed using SPSS 18 and descriptive statistics (determining frequency, percentage, mean and standard deviation) and analytical statistics (Chi-square test, Pearson’s correlation coefficient test and linear regression). In analyzing the data, *P*-value less than 0.05 was considered statistically significant.

## Results

In this study, the nurses were between 27 and 48 years of age, with an average age of 34 ± 3.8. In terms of work experience, the lowest and highest experiences were 2 and 21 years, with an average of 12 ± 2.5 years.

In this study, the majority of the research participants were female (182, 89.2%) and married (178, 87.2%). Other demographic findings are presented in Table1. 

**Table1:** Distribution of demographic data

Demographic Data		Number	Percentage
Age (year)	Less than 35	46	22.6
	More than 35	158	77.4
Job Experience (year)	Less than 10	30	14.7
	More than 10	174	85.3
Education Level	Bachelor’s degree	192	94.1
	Master’s degree	12	5.9
Shift Work	Fixed	24	11.8
	Rotational	180	88.2
Ward	Internal	42	20.6
	Surgical	48	23.5
	Pediatric	40	19.6
	Emergency	34	16.7
	Intensive Care	40	19.6

The average score for the moral sensitivity of the samples was 5.70 ± 69.15, and 4.76 ± 20.01 for their self-esteem. Pearson’s correlation coefficient test indicated a significant and positive relationship between the two variables under study (r = 0.472 and *P* = 0.001) (Table 2). 

**Table2:** Mean score and standard deviation of moral sensitivity and self-esteem in nursing personnel

**Variable**	**Mean**	**Standard ** **Deviation**
Moral sensitivity	69.15	5.70
Self-Esteem	20.01	4.76
Pearson’s correlation coefficient test: *P* = 0.001, r = 0.472		

The distribution of moral sensitivity and self-esteem is represented in Table 3. Table 4 illustrates the relationship between demographic profiles and the two main variables according to the linear regression test. In the data analysis, a *P*-value less than 0.05 was considered statistically significant.

**Table3:** Distribution of moral sensitivity and self-esteem in nursing personnel

Self-Esteem	High	Moderate	Low	Total
Moral Sensitivity				
Severe	20 (9.8%)	12 (5.9%)	0 (0%)	32 (15.7%)
Moderate	30 (14.7%)	125 (61.2%)	2 (1%)	157 (76.9%)
Mild	0 (0%)	8 (3.9%)	7 (3.5%)	15 (7.4%)
Total	50 (24.5%)	145 (71%)	9 (4.5%)	204 (100%)

**Table4:** Relationship between demographic data and main variables

Main Variable	Moral Sensitivity		Self-Esteem	
Demographic Data	P	R	P	R
Gender	0.190	0.063	0.245	0.082
Marital Status	0.115	0.045	0.189	0.055
Age	0.137	0.073	0.294	0.062
Work Experience	0.156	0.046	0.188	0.074
Education Level	0.148	0.081	*0.001	0.033
Shift Work	0.196	0.018	0.264	0.057
Ward	0.162	0.022	0.144	0.028

## Discussion

The results of the study revealed that there is a positive and significant correlation between the moral sensitivity and self-esteem variables among nurses. In other words, nurses who enjoy a higher moral sensitivity have greater self-esteem. Although we could find no studies in the available literature on the correlation between the two variables of sensitivity and self-esteem specifically, the relationship between other moral variables and self-esteem has been investigated. The results of one study in the United States suggested that high self-esteem in nurses would nurture a positive understanding of personal dignity, which is an ethical concept (13). Similarly, another study in the United States illustrated that as nurses understand their professional values, their self-esteem increases (24). In this regard, the findings of a study in Iran indicated that the perceived support as a moral variable increases nurses’ self-esteem in the workplace (25). In another study the relationship between moral sensitivity and psychosocial work environment factors was measured in Iranian nurses, and significant correlations between the psychosocial work environment factors and the moral sensitivity in nurses was found (26). The findings of the present study and the studies mentioned above confirm the fact that moral variables can be related to psychological variables.

Current study results showed that the moral sensitivity score of the majority of the nurses under study was of moderate level. These findings align with those obtained from other relevant studies as well (5 - 6, 20, 27).

Studies conducted in the area of moral sensitivity suggest that this moral trait stands as a cognitive ability for nurses (28) and is useful for both patients and nurses, in that it can promote the quality of patient care through encouraging cooperation with health professionals (1). Also, the results of one study in Sweden revealed that nurses with higher moral sensitivity experience less moral stress (4). Research findings in Iran indicated that enhanced moral sensitivity in nurses leads to job satisfaction (26), and the results of a research in Korea also showed that nurses who possessed higher moral sensitivity scored better on the application of the code of ethics (6). In this regard, the results of studies conducted in Iran on moral sensitivity in the nursing profession are negotiable. Iranian researchers have demonstrated that moral sensitivity is a part of the professional competence of nurses and improves their moral performance (29). Other Iranian researchers have identified the concept of moral sensitivity in nursing as containing elements such as sensitivity to care, sensitivity to work errors and sensitivity in care decisions (8). In addition, another study suggested that having high moral sensitivity in nurses leads to an increase in moral self-concept (5). Overall, studies that conceptually evaluate moral sensitivity in nurses emphasize the need to improve the level of moral sensitivity in this particular group of medical staff.

Since ethical issues may spell trouble in the workplace at the individual, organizational and societal levels (1), and nurses need moral knowledge in order to cope with a number of ethical challenges, high moral sensitivity can further help them in this regard. Therefore, with respect to the merits of developing high moral sensitivity in nurses, the concept of moral sensitivity in the nursing profession should be considered more closely, and nurses need to be trained up from medium to high level by professors of clinical ethics. Incidentally, a study in China demonstrated that lack of ethical knowledge in nurses prevents the promotion of moral sensitivity in the clinic (30). Likewise, paying attention to the moral sensitivity of other members of the healthcare team such as physicians has been a topic of research. While the moral sensitivity of nurses was moderate in this study, another research in Iran found a high level of moral sensitivity in physicians using the same questionnaire (31). The reason for the higher level of moral sensitivity in physicians can be attributed to their difficult professional conditions. In fact, doctors carry out their duties in an atmosphere that requires high moral sensitivity in patient-related therapeutic decision-making, especially in critical situations. As a result, it can be stated that physicians have higher moral sensitivity levels than nurses. Therefore, it seems necessary to consider ethical education measures for nurses in order to achieve a more satisfactory level of moral sensitivity. 

The present study also showed the self-esteem of the majority of nurses to be at a moderate level. Similarly, the results of a study in Iran also indicated that the majority of hospital staff had moderate self-esteem, and the higher the self-esteem of the staff was, the higher their organizational commitment would be (32). The researchers’ clinical experiences suggest that self-esteem in nursing is interspersed with concepts such as the feeling of self-worth, professional socialization and understanding professionalism; and that high self-esteem serves as a basis for improved professional performance. The findings of other studies set forth the advantages of high self-esteem for nurses. According to the results of a research in South Korea, self-esteem is one of the factors that help nurses develop clinical skills (33). A research in Nepal also indicated that high self-esteem in nurses is associated with a more effective use of coping strategies (34).

The enhanced efficiency resulting from high self-esteem on the part of nurses has been explicitly stated in other studies. Research in Taiwan illustrated that it promotes greater job satisfaction and thus bolsters a sustained career (8). Similarly, a study in China demonstrated that high self-esteem in nurses encourages them to stay at their jobs and gain more professional success (35). A study in Iran also showed that high self-esteem would add to nurses’ professional independence (36).

A broad range of benefits resulting from high self-esteem in nurses reminds us that this important personality trait needs to be raised from medium to high; otherwise, nurses may be plagued with a problem that might cause their low level of self-esteem to decline even further. With respect to this dilemma, studies have been conducted whose results could worry the authorities. Nurses with low self-esteem have experienced bullying to a great degree in Spain (11), and in Taiwan, they have not attained the required emotional sophistication in their work place and self-sabotaging behavior tends to be extremely high (12). Therefore, it is essential to enhance self-esteem in nurses by implementing educational and psychological interventions.

The findings of the research did not yield any relationship between the demographic data of the research participants and the moral sensitivity variable. In terms of self-esteem, there was a positive and significant relationship only between education level and this variable, so that the higher the nurses’ degree was, the higher their self-esteem would be. Similar findings were obtained about nurses in Germany as well (21). It can be inferred that promoting the continuation of advanced education among the nursing staff will contribute to their improved self-esteem. 

It should be mentioned that there were limitations to this research, for instance we were unable to get all the distributed questionnaires back, and some that we did were defaced. Another issue, which was beyond our control, was that the nurses could have developed different perceptions of the main variables of the study at the time they were completing the questionnaires due to specific conditions encountered in the workplace. Furthermore, we could not find any studies on the correlation between the two variables of moral sensitivity and self-esteem, and therefore it was impossible to compare and contrast the findings of the current study with other similar works. 

## Conclusion

Considering the medium level of moral sensitivity and self-esteem in the majority of the participants in this study, it seems necessary to focus on the moral and personal characteristics of nurses. Based on the results of this study, there is a significant and positive relationship between moral sensitivity and self-esteem in nurses. Consequently, in order to strengthen the moral sensitivity of nurses, it is recommended to implement psychological educational interventions focusing on improving their self-esteem. Other interventions may include implementing strategies such as periodic and regular training in the field of clinical ethics, and improving nurses’ knowledge on clinical ethics. In addition, current study results will help nursing managers and hospital authorities plan and offer training courses to promote nurses’ moral sensitivity and self-esteem, which will eventually lead to empowering them both morally and psychologically, and providing quality care to patients.
